# Resveratrol attenuates chronic pulmonary embolism-related endothelial cell injury by modulating oxidative stress, inflammation, and autophagy

**DOI:** 10.1016/j.clinsp.2022.100083

**Published:** 2022-08-03

**Authors:** Xiaopeng Liu, Haiying Zhou, Zhixiong Hu

**Affiliations:** Department of Respiratory Medicine, Jinshan Hospital Affiliated to Fudan University, Shanghai, China

**Keywords:** Pulmonary artery endothelial dysfunction, Chronic thromboembolic pulmonary hypertension, Oxidative stress, Inflammation, Autophagy, Resveratrol

## Abstract

**Objectives:**

Due to Pulmonary Artery Endothelial Cell (PAEC) dysfunction, Pulmonary Hypertension (PH) persists even after the Pulmonary Embolism (PE) has been relieved. However, the mechanism behind this remains unclear.

**Method:**

Here, the authors incubated Human PAECs (HPAECs) with thrombin to simulate the process of arterial thrombosis.

**Results:**

CCK8 results showed a decrease in the viability of HPAECs after thrombin incubation. In addition, the expression of Tissue Factor (TF), Monocyte Chemoattractant Protein 1 (MCP-1), VCAM-1, ICAM-1, cleaved caspase 3, cleaved caspase 9, and Bax protein were all increased after thrombin incubation, while Bcl-2 was decreased. The effects of 3-MA treatment further suggested that autophagy might mediate the partial protective effects of Resveratrol on HPAECs. To observe the effects of Resveratrol *in vivo*, the authors established a Chronic Thromboembolic Pulmonary Hypertension (CTEPH) model by repeatedly injecting autologous blood clots into a rat's left jugular vein. The results exhibited that Mean Pulmonary Arterial Pressure (mPAP) and vessel Wall Area/Total Area (WA/TA) ratio were both decreased after Resveratrol treatment. Moreover, Resveratrol could reduce the concentration and activity of TF, vWF, P-selectin, and promote these Superoxide Dismutase (SOD) in plasma. Western blot analysis of inflammation, platelet activation, autophagy, and apoptosis-associated proteins in pulmonary artery tissue validated the results in PHAECs.

**Conclusions:**

These findings suggested that reduced autophagy, increased oxidative stress, increased platelet activation, and increased inflammation were involved in CTEPH-induced HPAEC dysfunction and the development of PH, while Resveratrol could improve PAEC dysfunction and PH.

## Highlights

• Resveratrol can effectively improve pulmonary thromboembolism-induced PAEC injury.
• Resveratrol can reduce pulmonary arterial pressure through a variety of mechanisms.
• These findings may contribute to the treatment of PH in the future.

## Introduction

Pulmonary Embolism (PE) has a very high mortality rate, ranking as the third most common cardiovascular disease.^1^ Clinical investigations have shown that PE carries a very poor prognosis, with a mortality rate of 13% and 26% within 1 month and 1 year of diagnosis, respectively, posing a serious threat to patients' lives.^2^ Chronic Thromboembolic Pulmonary Hypertension (CTEPH) is a chronic development of Pulmonary Hypertension (PH) in the pulmonary arteries and their branches due to thromboembolism, resulting in long-term obstruction of blood flow, and consequent intimal thickening and remodeling of the small pulmonary arteries.^3^^,^^4^ Impaired Pulmonary Artery Endothelial Cells (PAECs) and abnormal function play an important role in this process.^5^^,^^6^ Olaf Mercier et al.^7^ suggested that PAECs in patients with CTEPH may induce Pulmonary Artery Smooth Muscle Cell (PASMC) growth and monocyte migration via paracrine of growth factors and cytokines, aggravating fibrotic vascular remodeling.

Resveratrol is a polyphenolic compound isolated from Veratrum grandiflorum with a variety of biological activities including antioxidant, anti-inflammatory, and anti-tumor properties.^8^ Resveratrol has been reported to improve PH by modulating multiple signaling pathways. For example, Resveratrol inhibits SphK1-mediated NF-κB activation and suppresses PASMC proliferation and pulmonary vessel muscularization.^9^ According to Ying-Ying Liu et al.,^10^ Resveratrol inhibits the proliferation of PASMCs by regulating the miR-638/NR4A3/cyclin D1 axis. Additionally, Resveratrol activates the SIRT1 pathway and regulates the expression of p21 and cyclin D1 in PASMCs.^11^ Resveratrol prevents PASMCs proliferation by inhibiting the PI3K/AKT pathway and hypoxia-induced arginase II expression.^12^ Except for its effect on proliferation, Resveratrol administration inhibits HIF-1 alpha expression *in vivo* and *in vitro*, suppressed peripulmonary artery inflammatory cell infiltration, and reduces hypoxia-induced Reactive Oxygen Species (ROS) production in PAMSC.^13^ In terms of effects on inflammation, Resveratrol is also reported to inhibit PE-induced MCP-1 expression and MAPK pathway and reduced mean pulmonary artery pressure (mPAP) in rats with PE.^14^ Moreover, Resveratrol inhibits the RhoA-ROCK signaling pathway, suppresses Th17 cell differentiation, and improves PH^15^.

Although the effects of Resveratrol on PE or PH are known, there are few available reports on the role of Resveratrol in PAECs. According to Bruder et al.,^16^ Resveratrol treatment produces an elongation of bovine PAECs in a dose-dependent manner. Moreover, Resveratrol also has anti-inflammatory effects on PAECs. For example, the study of Jian-Wei Lin et al.^17^ showed that Resveratrol down-regulated TNF-α-induced MCP-1 level in primary rat PAECs via the MAPK signaling pathway. Besides, Resveratrol and its metabolites improve HAECs damage and inflammation, manifested by reduced eotaxin-1 responses in atherosclerosis.^18^ Except for anti-inflammatory effects, Resveratrol also significantly improved endothelial dysfunction and attenuated oxidative stress and NADPH oxidase expression in small pulmonary arteries induced by monocrotaline in rats.^19^ However, the role of Resveratrol in PAECs has not been studied in the CTEPH model or in human-derived PAECs.

In this study, by incubating Human PAECs (HPAECs) with thrombin and repeatedly injecting autologous blood clots into rat left jugular vein, the authors investigated the effects of Resveratrol on CTEPH *in vitro* and *in vivo*. Through detecting the expression of inflammatory cytokines, chemokines, SOD, platelet activation markers, autophagy-related proteins, and apoptosis-related proteins in HPAECs and pulmonary artery tissues, the authors explored the possible mechanisms by which Resveratrol protected PAECs to provide a basis for the clinical application of Resveratrol.

## Materials and methods

### HPAEC culture

HPAECs (ATCC, PCS-100-022, Manassas, VA, USA) were cultured in Endothelial Basal Medium 2 (EBM2) containing 10% FBS, 100 U/mL penicillin, and 100 U/mL streptomycins at 37°C with 5% CO_2_. After growth to confluence, HPAECs were incubated in the same serum-free EBM2 medium for 1‒2 hours, stimulated with thrombin that mimics the chronic thrombotic stimulation process in CTEPH, and incubated with various concentrations of RSV (5, 10 or 20 μM, Sigma-Aldrich, St. Louis, MO, USA). Cell assays were done on HPAECs within passages 7.

### CTEPH model

Twenty-seven male Sprague Dawley rats (Shanghai SLAC Laboratory Animal Corporation, Shanghai, China), weighing 250‒300 g and 3 months old, were housed in an air-conditioned room at 23 ± 2°C and 65 ± 5% humidity, with free access to food and water. This study was approved by the Ethics Committee of Jinshan Hospital Affiliated with Fudan University. The rats were randomly divided into Sham, CTEPH, and CTEPH+RSV groups, and each group was divided into three subgroups according to the timing of observation (1 week, 2 weeks and 4 weeks), with 3 rats in each group. Autologous blood clots were prepared and CTEPH models were established according to a previous study.^20^ RSV (10 mg/kg/day) was intraperitoneally injected to the rats for 1h prior to the start of the CTEPH protocol.

### Cell counting kit-8 (CCK8) assay

HPAEC viability was assessed using CCK8 (Beyotime C0037, Wuhan, China) according to the manufacturer's protocol. Briefly, a total of 1 × 10^4^ cells/well were inoculated in 96-well plates. After thrombin treatment, 10 µL of CCK8 solution was added to the medium and incubated at 37°C and 5% CO_2_ for 2h. Absorbance at 6h, 12h, and 24h was measured at 450 nm using an Eon spectrophotometer (BioTek Instruments, Winooski, VT, USA). The experiments were performed independently in triplicate.

### Western blot

After lysis of HPAECs or pulmonary artery tissues, protein concentrations were determined by the BCA method. Tissue Factor (TF), MCP-1, Acetylated-Forkhead box O1 (Ac-FOXO1), FOXO1, VCAM-1, ICAM-1, caspase 3, caspase 9, cleaved caspase 3, cleaved caspase 9, Bax, Bcl-2, p62, LC3, von Willebrand Factor (vWF), and P-selectin protein expression were analyzed by Western blot analysis according to a previous study.^21^ For the detection of target proteins in cells, rabbit anti-human primary antibody (Santa Cruz Biotechnology, Dallas, TX, USA) was used. For detection of target proteins in tissues, rabbit anti-rat primary antibody (Abcam, Shanghai, China) was used. After incubation of primary antibody overnight at 4°C, membranes were incubated with goat anti-rabbit secondary antibody (1:5000, Abcam) for 2h.

### Enzyme-linked immunosorbent assay (ELISA) and activity assay

After the rats were anesthetized, blood was taken from the ophthalmic vein plexus and left to stand at 4°C. Then, the supernatant was collected by centrifugation. IL-6, IL-1β, TF, MCP-1, MPO, SOD, vWF, P-selectin, VCAM-1, ICAM-1 level in rat peripheral blood and HPAECs culture supernatants were determined as per the instructions of the ELISA kits (R&D Systems, Minneapolis, MN, USA). Optical Density (OD) at 450 nm was recorded using an Eon spectrophotometer (BioTek Instruments, Winooski, VT, USA). Experiments were performed independently in triplicate. Colorimetric activity assays were performed to determine plasma TF (Abcam), MPO (Sigma-Aldrich), SOD (Sigma-Aldrich), and vWF activity (Abcam) in rats at weeks 1, 2 and 4, respectively, according to the manufacturer's instructions.

### ROS measurement

The ROS assay was performed as previously described.^22^ Briefly, 1 × 10^6^ cells were incubated with 10 µmoL/L DCF-DA (Sigma) at 37°C for 30 min and cell fluorescence intensity was recorded by FACS Canto flow cytometer and analyzed using FlowJo Software (Tree Star Inc, OR, USA).

### mPAP measurement

The mPAP was recorded according to the previous method.^14^ After repeated injections of autologous blood clots at 1w, 2w and 4w respectively, rats were anesthetized with 10% chloral hydrate (0.3 g/kg). A polyvinyl chloride catheter was slowly inserted into the right external jugular vein and connected to a biosignal recorder. The mPAP was recorded when the catheter reached the pulmonary artery.

### Histological analysis

According to a previous report, lungs were dissected, and pulmonary artery tissue was extracted.^23^ Lung tissues were fixed in 10% formaldehyde for 24h, paraffin-embedded, and sectioned at 5 μm thickness. Hematoxylin-Eosin (HE) staining was performed according to a previous method.^21^ Pulmonary artery pathological changes were observed by light microscopy (BX5, Olympus, Tokyo, Japan), and the degree of pulmonary artery remodeling was evaluated by calculating the Wall Area/Total Area (WA/TA) ratio of pulmonary artery vessel using ImageJ software (NIH, Bethesda, MD).

### Statistical analyses

Data were analyzed using GraphPad Prism 9.0.0 software (GraphPad Software, San Diego, CA, USA) and expressed as mean ± standard deviation. One-way analysis of variance (ANOVA) or unpaired Student's *t*-test was used for statistical analysis of differences between groups, with a p-value < 0.05 considered a significant difference.

## Results

### Thrombin promoted HPAEC inflammatory injury

**Fig. 1:**
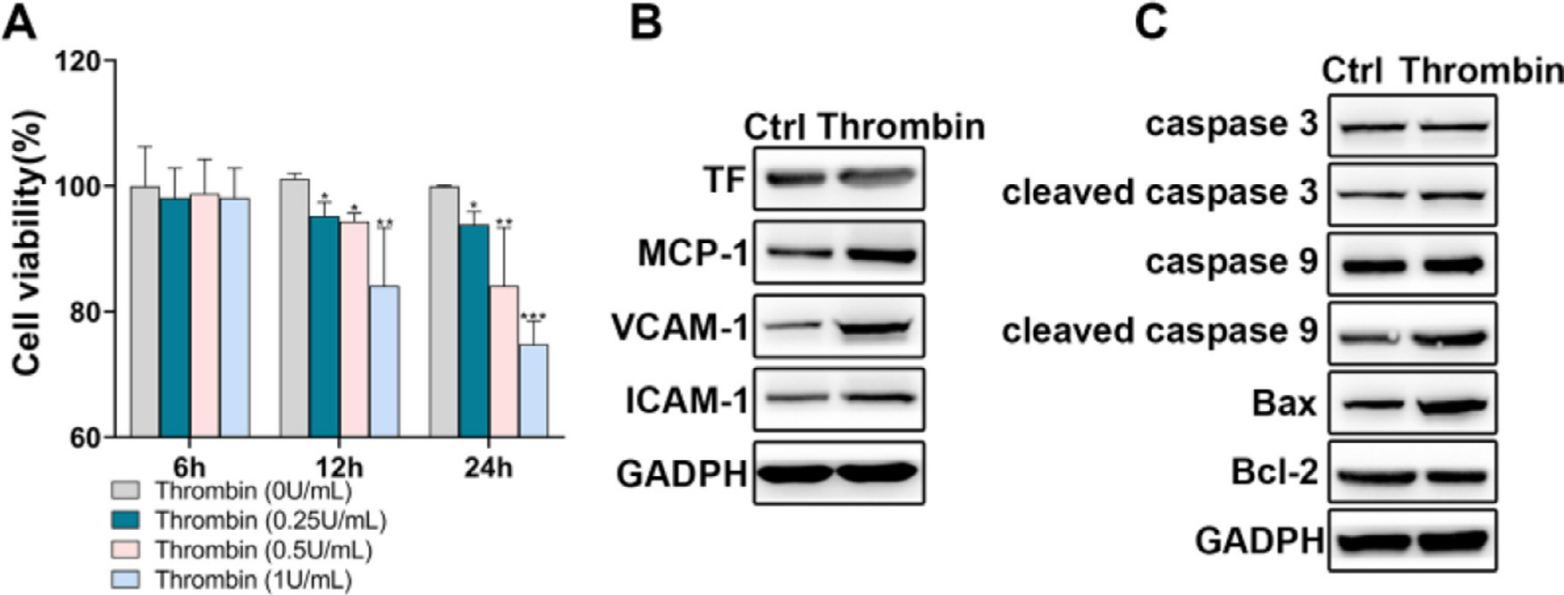
HPAECs were damaged upon thrombin stimulation. A, HPAEC viability upon thrombin stimulation was detected by CCK-8. B, Western blot analysis of TF, MCP-1, VCAM-1, and ICAM-1 in HPAECs. C, Western blot analysis of caspase 3, caspase 9, cleaved caspase 3, cleaved caspase 9, Bax, and Bcl-2 in HPAECs. * p < 0.05; ** p < 0.01; *** p < 0.001. All experiments were repeated three times.

The authors first incubated HPAECs with thrombin to simulate the process of PE. CCK-8 assay showed that thrombin inhibited HPAEC viability in a time-dependent and concentration-dependent manner (Fig. 1A). Thrombin of 0.5 U/mL and 12h incubation duration was chosen for subsequent experiments. The authors then examined the effects of thrombin on the expression of TF, chemokines, and adhesion molecules in HPAECs. The results showed that thrombin promoted the expression of TF, MCP-1, VCAM-1, and ICAM-1 (Fig. 1B). In addition, Western blot analysis of apoptosis-related proteins also showed that thrombin could promote apoptosis in HPAECs (Fig. 1C).

### Resveratrol improved thrombin-induced HPAEC dysfunction

**Fig. 2:**
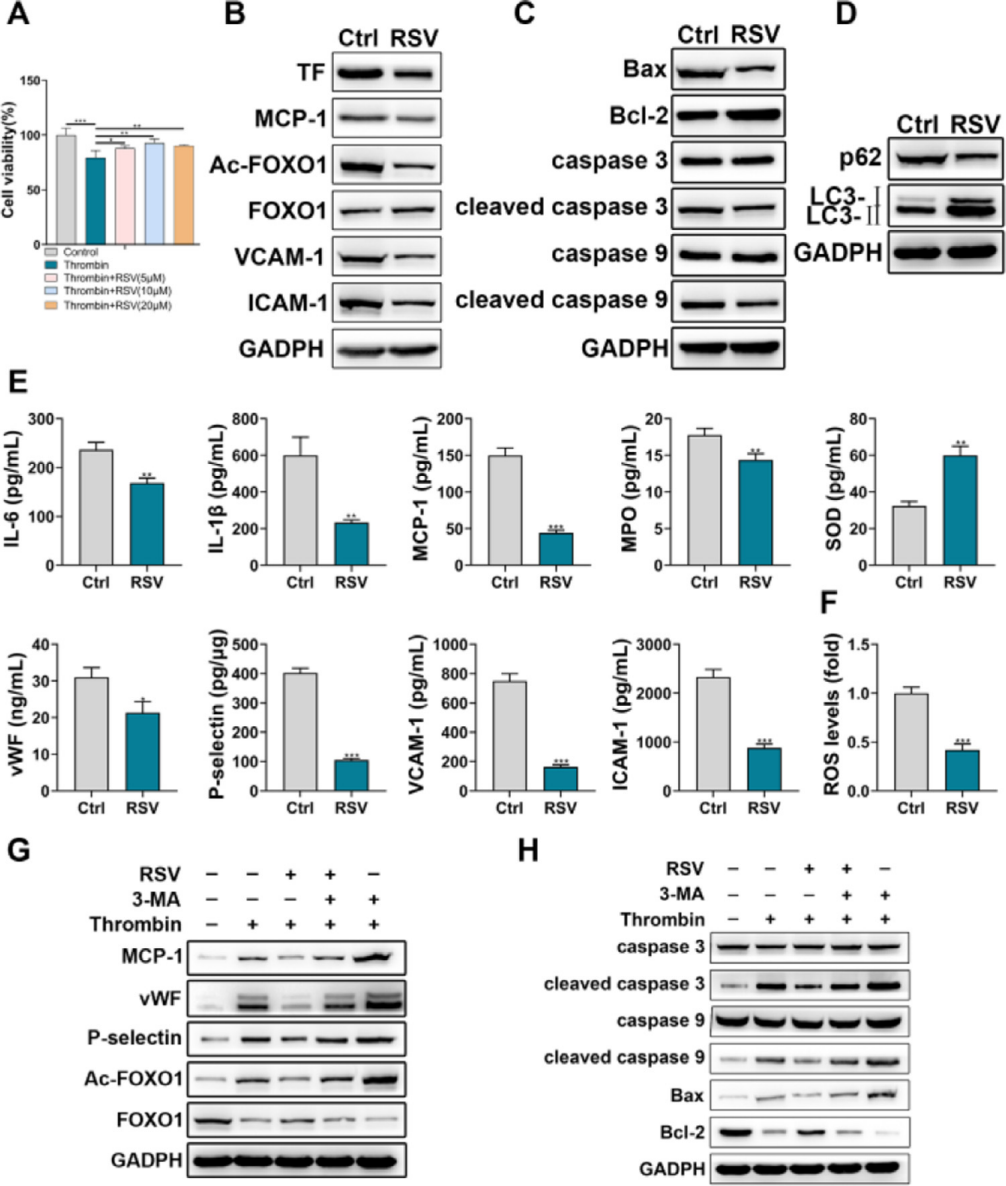
Resveratrol improved HPAECs damage. A, The viability of HPAECs treated with Resveratrol was detected by CCK-8. B, Western blot analysis of TF, MCP-1, Ac-FOXO1, FOXO1, VCAM-1, ICAM-1 in HPAECs treated with Resveratrol. C, Western blot analysis of caspase 3, caspase 9, cleaved caspase 3, cleaved caspase 9, Bax, and Bcl-2 in HPAECs treated with Resveratrol. D, Western blot analysis of p62 and LC3 in HPAECs treated with Resveratrol. E, IL-6, IL-1β, MCP-1, MPO, SOD, vWF, P-selectin, VCAM-1, and ICAM-1 in HPAEC culture supernatant were assessed by ELISA. F, ROS in HPAECs was measured by flow cytometry. G, Western blot analysis of MCP-1, vWF, P-selectin, Ac-FOXO1, and FOXO1 in HPAECs treated with Resveratrol or 3-MA. H, Western blot analysis of caspase 3, caspase 9, cleaved caspase 3, cleaved caspase 9, Bax, and Bcl-2 in HPAECs treated with Resveratrol or 3-MA.

The effects of Resveratrol on HPAEC dysfunction were investigated via multiple assays. CCK-8 assay showed that Resveratrol could partially restore the thrombin-induced decrease in HPAEC viability (Fig. 2A). In addition, Western blot results showed that Resveratrol could inhibit the expression of TF, MCP-1, Ac-FOXO1, VCAM-1, and ICAM-1 (Fig. 2B). The detection of apoptosis-related and autophagy-related proteins revealed that Resveratrol could inhibit apoptosis of HPAEC (Fig. 2C) and promote autophagy of HPAEC (Fig. 2D). Moreover, the authors examined the effects of Resveratrol on inflammation and injury using ELISA. The results showed that Resveratrol could inhibit the expression of IL-6, IL-1β, MCP-1, MPO, vWF, P-selectin, VCAM-1, ICAM-1 and promote the expression of SOD (Fig. 2E). Meanwhile, flow cytometry results showed that Resveratrol could scavenge ROS to some extent (Fig. 2F). Besides, through western blot, the authors found that the inhibitory effect of Resveratrol on MCP-1, vWF, P-selectin, Ac-FOXO1, cleaved caspase 3, cleaved caspase 9, and Bax was partially reversed by 3-MA (Fig. 2G, H).

### Resveratrol inhibited the general level of inflammation, oxidative stress, and platelet activation in CTEPH rats

**Fig. 3:**
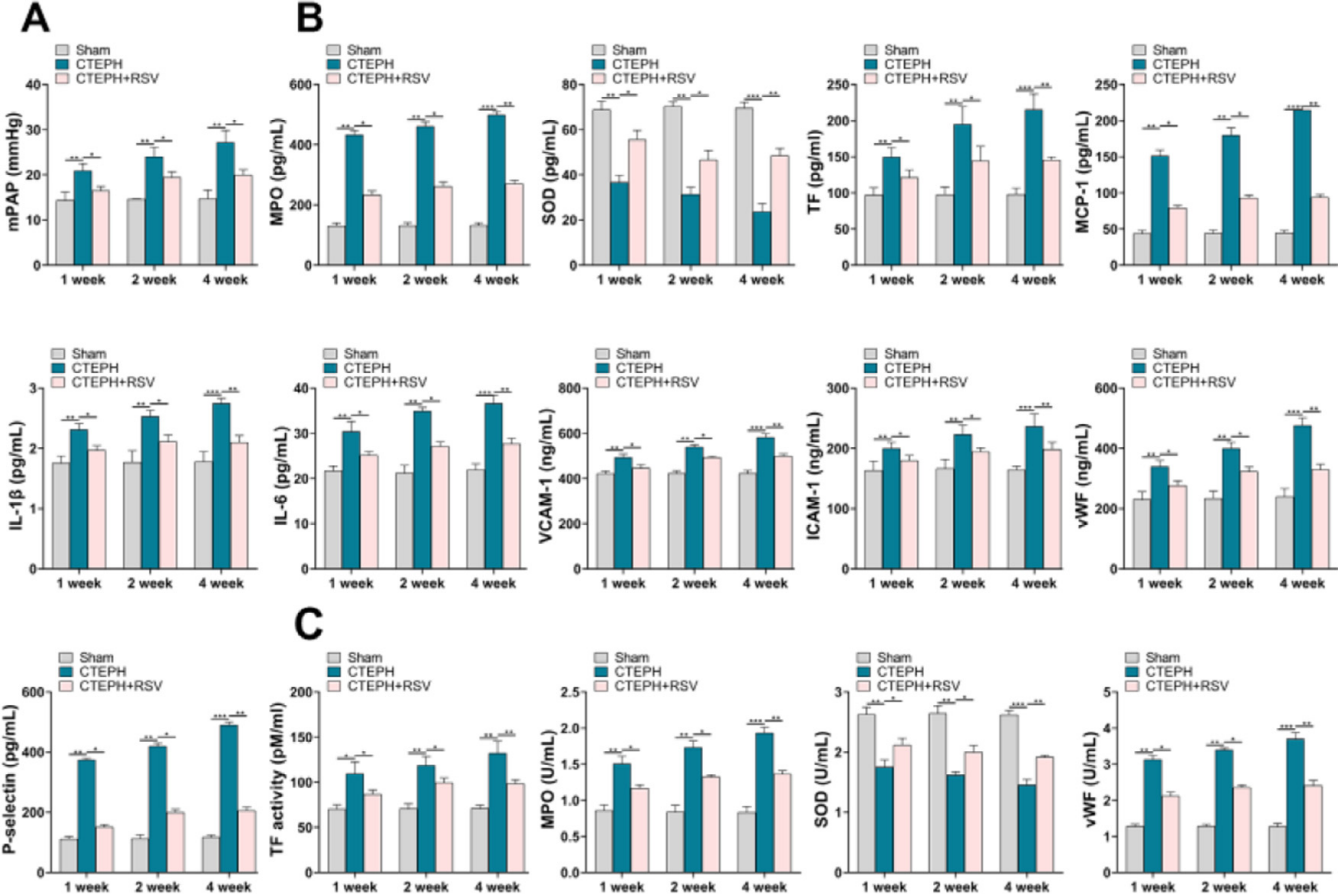
Resveratrol lowered mPAP and altered plasma cytokine profiles. A, mPAP was recorded in CTEPH rats. B, ELISA assay for concentrations of MPO, SOD, TF, MCP-1, IL-1β, IL-6, VCAM-1, ICAM-1, vWF, and P-selectin in peripheral blood of CTEPH rats. C, The activity of TF, MPO, SOD and vWF in peripheral blood was measured.

The role of Resveratrol in vivo was investigated in a CTEPH rat model. As shown in Fig. 3A, elevated mPAP in CTEPH rats could be reduced by Resveratrol. This change occurred from the first week onwards. ELISA results showed that Resveratrol was able to downregulate the levels of MPO, TF, MCP-1, IL-1β, IL-6, VCAM-1, ICAM-1, vWF, P-selectin and increase the level of SOD in peripheral blood (Fig. 3B). In addition, the activity of TF, MPO, and vWF in peripheral blood decreased accordingly after Resveratrol treatment, while SOD activity increased (Fig. 3C).

### Resveratrol alleviated inflammatory damage to the pulmonary arteries in CTEPH rats

**Fig. 4:**
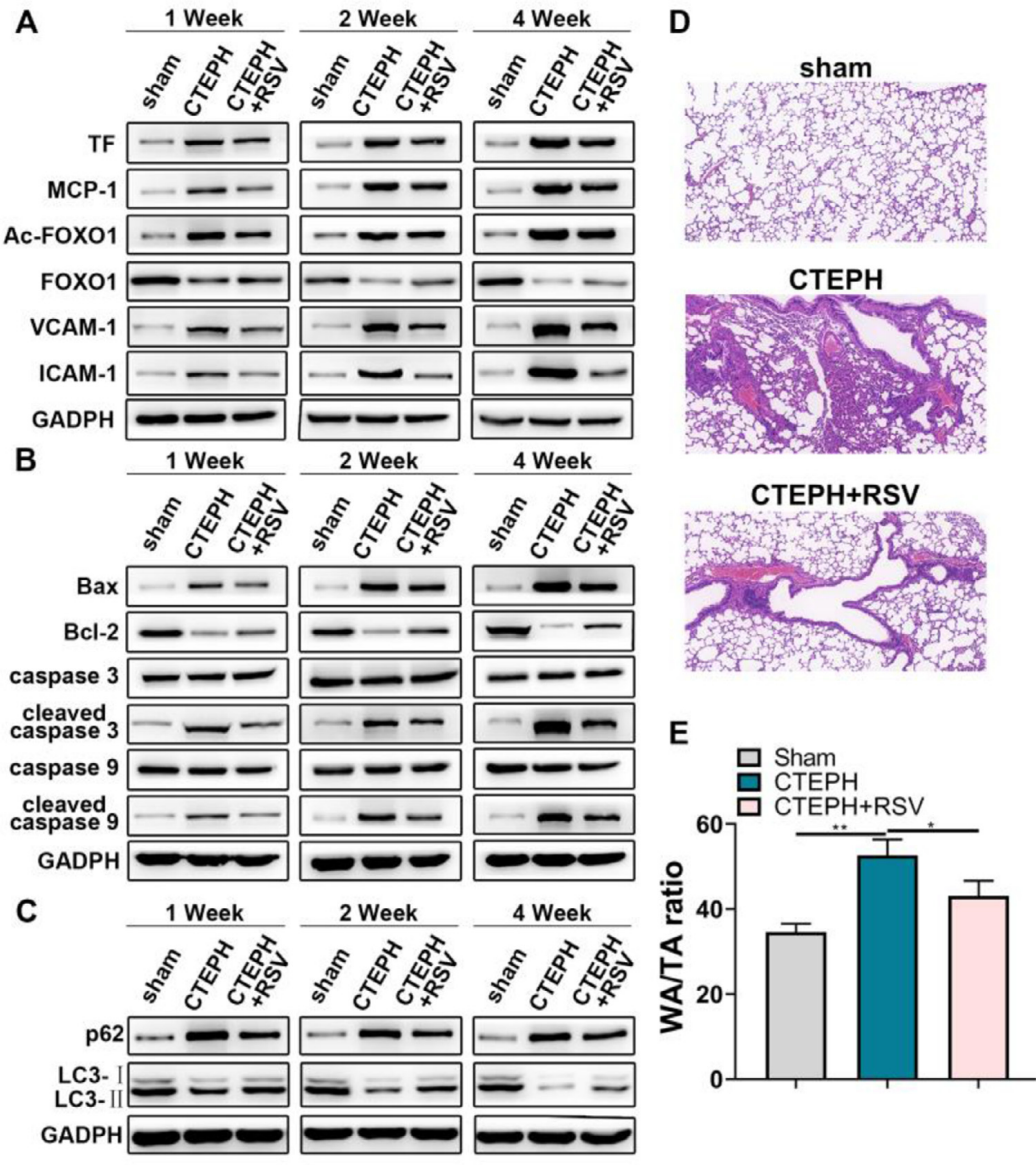
Resveratrol promoted thrombolysis and pulmonary artery function recovery. A, Western blot assay for TF, MCP-1, Ac-FOXO1, FOXO1, VCAM-1, and ICAM-1 expression in pulmonary artery tissues. B, Western blot assay for caspase 3, caspase 9, cleaved caspase 3, cleaved caspase 9, Bax and Bcl-2 expression in pulmonary artery tissues. C, Western blot assay for p62 and LC3 expression in pulmonary artery tissues. D, HE staining for pulmonary tissues. E, WA/TA ratio was measured.

Western blot analysis was performed in dissected pulmonary artery tissues. The results showed that Resveratrol could inhibit the expression of TF, MCP-1, Ac-FOXO1, VCAM-1 and ICAM-1 (Fig. 4A). After Resveratrol treatment, the protein expression of pro-apoptotic and p62 decreased, while the expression of LC3-Ⅱ protein increased (Fig. 4B, C). In addition, HE is staining of lung tissues revealed that Resveratrol alleviated CTEPH-induced thrombosis and pulmonary artery wall thickening (Fig. 4D, E).

## Discussion

PAEC dysfunction contributes to CTEPH progression. Here, the authors found that Resveratrol ameliorated thrombin-induced HPAEC cell apoptosis, which might be related to the inhibition of inflammatory response, platelet activation, oxidative stress, and promotion of autophagy by Resveratrol. In a CTEPH rat model, the authors further verified the effects of Resveratrol on pulmonary arteries. The authors found that Resveratrol reduced mPAP, as well as decreased the levels of inflammatory cytokines, chemokines, and adhesion molecules in peripheral blood increasing the levels of SOD. Additionally, Resveratrol could inhibit platelet activation. In pulmonary artery tissues, Resveratrol treatment resulted in decreased levels of apoptosis, increased levels of autophagy, and improvement in CTEPH-induced thrombus formation and thickened pulmonary artery walls. These findings suggested that Resveratrol might be a promising medication for CTEPH.

Resveratrol is a naturally non-flavonoid polyphenol,^24^ abundant in wine, berries and peanuts.^8^ It has a plasma half-life of only 8 to 14 minutes^25^ and peaks in plasma after 1 hour after ingestion.^26^ Many studies have labeled Resveratrol as an antioxidant, anti-platelet activator or anti-inflammatory agent that could play a potential therapeutic role in cardiovascular disease by scavenging ROS in vivo, inhibiting cyclooxygenases, and activating many anti-inflammatory pathways.^27^ For example, Resveratrol increased serum concentrations of SIRT1, thereby inhibiting NF-κB signaling pathway activation and the synthesis of pro-inflammatory cytokines.^28^ Rivera et al. showed that Resveratrol could induce lipocalin expression and improved cardiometabolic disorders.^29^ A derivative of Resveratrol helped to release nitric oxide and inhibit platelet aggregation via the arachidonic acid agonist pathway.^30^ Park et al.^31^ described Resveratrol as a potent antagonist of phosphodiesterase, reducing the contractile response of vascular smooth muscle cells in a PDE1-dependent manner and alleviating hypertension. Moreover, Resveratrol might reduce insulin resistance by decreasing the expression of enzymes responsible for free radical production and increasing the production of enzymes involved in scavenging ROS.^32^ Resveratrol as a natural autophagy regulator has a therapeutic role in many diseases. For example, it could prevent and treat Alzheimer's Disease by regulating the mTOR signal pathway, activating SIRT1, and deacetylating histone acetylases.^33^ Additionally, Resveratrol has neuroprotective effects through modulation of autophagy- and inflammation-related pathway to reduce cerebral ischemic injury.^34^ Moreover, Resveratrol prevents post-ovulatory oocyte senescence by inducing mitochondrial autophagy.^35^ However, the effects of Resveratrol on PAECs in CTEPH have not been reported. Whether it ameliorates CTEPH-induced PAEC dysfunction by modulating autophagy has also never been studied.

In this study, to investigate the roles of Resveratrol in CTEPH, the authors tested a number of biochemical parameters. Considering that inflammation is a possible driver of CTEPH progression,^36^^,^^37^ inflammatory mediators were measured. MCP-1 is a key mediator in stimulating the infiltration of inflammatory cells into the lung, reported being expressed in all pulmonary artery walls after pulmonary thromboembolism.^17^ VCAM-1 and ICAM-1 are inflammatory mediators secreted by endothelial cells that promote the infiltration of inflammatory cells into the pulmonary artery tissue.^38^ MPO, a marker of neutrophil activation, is enriched in neutrophils and released upon external stimuli.^39^ Besides, TF is a kind of pro-coagulant whose upregulated expression plays a key role in thrombosis.^40^ SOD, also called superoxide dismutase, controls the levels of various ROS and reactive nitrogen, thereby limiting the potential toxicity of these molecules.^41^ Furthermore, vWF and P-selectin were also measured. When endothelial cells are damaged, they may secrete vWF into the circulation, which binds to circulating platelets to form emboli and mediates the recruitment of leukocytes to the vascular endothelial surface along with P-selectin.^42^ The dysregulation of these biochemical parameters and effectiveness of Resveratrol suggested the importance of inflammation in driving PH progression and opens up new prospects for more targeted treatment. The present results implied that current or new immunosuppressive agents targeting inflammation might be promising drugs for CTEPH. However, the clinical application of these drugs should be following careful validation of safety and effectiveness.

In conclusion, by constructing *in vitro* and *in vivo* models of PE, the authors found that Resveratrol can effectively improve pulmonary thromboembolism-induced PAEC injury and reduce pulmonary arterial pressure through a variety of mechanisms, including anti-inflammatory, anti-oxidant, anti-coagulant and pro-autophagy effects.

## Authors’ contributions

Xiaopeng Liu, Haiying Zhou and Zhixiong Hu designed experiments, carried out experiments, and analyzed experimental results. Xiaopeng Liu wrote the manuscript, Zhixiong Hu revised the manuscript. All authors approved the final manuscript.

## Funding

This study was supported by the grants from the Cultivation Plan for “Outstanding Young Talented” in the Fourth Cycle of Health System of Jinshan District, Shanghai (n° JSYQ201907).

## Conflicts of interest

The authors declare no conflicts of interest.
